# Chaotic Resonance in Typical Routes to Chaos in the Izhikevich Neuron Model

**DOI:** 10.1038/s41598-017-01511-y

**Published:** 2017-05-02

**Authors:** Sou Nobukawa, Haruhiko Nishimura, Teruya Yamanishi

**Affiliations:** 1 Department of Computer Science, Chiba Institute of Technology, 2-17-1 Tsudanuma, Narashino, 275-0016 Japan; 2 Graduate School of Applied Informatics, University of Hyogo, 7–1–28 Chuo-ku, Kobe, 650–8588 Japan; 3 Department of Management Information Science, Fukui University of Technology, 3-6-1 Gakuen, Fukui, 910-8505 Japan

## Abstract

Chaotic resonance (CR), in which a system responds to a weak signal through the effects of chaotic activities, is a known function of chaos in neural systems. The current belief suggests that chaotic states are induced by different routes to chaos in spiking neural systems. However, few studies have compared the efficiency of signal responses in CR across the different chaotic states in spiking neural systems. We focused herein on the Izhikevich neuron model, comparing the characteristics of CR in the chaotic states arising through the period-doubling or tangent bifurcation routes. We found that the signal response in CR had a unimodal maximum with respect to the stability of chaotic orbits in the tested chaotic states. Furthermore, the efficiency of signal responses at the edge of chaos became especially high as a result of synchronization between the input signal and the periodic component in chaotic spiking activity.

## Introduction

Stochastic resonance (SR) is a phenomenon in which the presence of noise helps a non-linear system amplify a weak (under-barrier) signal^1, 2^. In the past few decades, a considerable number of studies about SR in biological systems has been conducted^3–6^. More recently, studies of SR have been conducted using neural systems which possess various kinds of spiking patterns and complex physiological network structures. For example, Perc and Marhl examined frequency locking due to additive noise in the resting state near the bifurcation point leading to the chaotic-burst spiking state^7^. Nobukawa and Nishimura demonstrated that spike-timing-dependent plasticity may be made efficient through the effect of SR in neural systems composed of three types of spiking patterns: regular spiking (RS), intrinsically bursting (IB) and chattering (CH)^8^. Wang *et al*. showed that multiple SRs, in which coherence measures of signal responses are maximized at multiple levels of noise strength, was observed in scale-free spiking neural networks with synaptic delay and pacemaker neurons^9^. Yilmaz *et al*. demonstrated that the presence of electrical synapses can enhance the efficiency of signal transmission in SR in the scale-free spiking neural network when including electrical and chemical synapses^10^. Teramae *et al*.^11^ showed that the spontaneous activity widely observed in actual cortical neural networks can be reproduced when incorporating SR. They noted this in the spiking neural network in which the strength of excitatory synaptic weights obeys a non-Gaussian, long-tailed, typically log-normal distribution. Also, many kinds of synchronization phenomena which are not restricted to SR, such as synchronization transition and chimera states, have been widely found in scale-free complex and physiological spiking neural networks with both delay and multiple structures^12–17^.

Furthermore, several studies have analyzed synchronization phenomena typified by chaos synchronization and phase synchronization among neurons, and with external input signals, in spiking neural networks with chaotic spiking activity^18–21^. Among these synchronization phenomena, it has been known that fluctuating activities in deterministic chaos cause a phenomenon that is similar to SR. In the corresponding phenomenon, called chaotic resonance (CR), the system responds to the weak input signal through engaging the effects of intrinsic chaotic activities under conditions in which no additive noise exists^2, 22^. Initially, CR was investigated using a one-dimensional cubic map and Chua’s circuit^23–27^, though more recently neural systems have been utilized^28–33^. In a previous study, we discovered that the signal response of CR in a spiking neural system has a unimodal maximum with respect to the degree of stability for chaotic orbits, as quantified by the maximum Lyapunov exponent^34^. That is, the appropriate chaotic behavior leads to the generation of spikes (i.e., exceeds the threshold) not at specific times, but at varying scattered times for each trial, as input signals. This frequency distribution of these spike timings against the input signal becomes congruent with the shape of the input signal.

A considerable number of studies have been conducted on chaos and bifurcation in spiking neural systems, generating model systems that include the Hodgkin-Huxley, FitzHugh-Nagumo, and Hindmarsh-Rose models^35^. In particular, the Izhikevich neuron model, as a hybrid spiking neuron model, combines a continuous spike generation mechanism and a discontinuous after-spike resetting process; thus, the model can induce many kinds of bifurcations, and reproduce almost all spiking activities observed in actual neural systems simply by tuning a few parameters^36^. In addition, the variety of reproduced spiking patterns is high in comparison with other spiking neuron models^37^.

The hybrid spiking neuron model is one of the piecewise-smooth dynamical systems, in which dynamics are switched according to the state of the system^38^. Saito and colleagues have conducted chaos/bifurcation analysis and circuit implementation against piecewise-constant dynamical systems, and piecewise-linear dynamical systems, as simplified versions of the piecewise-smooth dynamical system^39–41^. In particular, Tsubone *et al*. proposed a systematic method to predict parameter regions for chaotic states using an analytical approach in the piecewise-constant dynamical system^41^. While in general, piecewise-smooth dynamical systems include non-linear terms similar to those seen in the Izhikevich neuron model, an approach for evaluating Lyapunov exponents and characteristic multipliers that considers the saltation matrix^38^ through simulations against exhaustive parameter sets is needed. On considering this approach, it is clear that this model has various kinds of bifurcations and routes to chaos when under the effect of the state-dependent jump in the resetting process^34, 42–44^. However, the signal responses of CR have not been evaluated in chaotic states produced through different routes.

In our preliminary work, we confirmed the presence of CR in chaotic states induced by different routes (i.e., the periodic-doubling bifurcation route and intermittency route to chaos) in the Izhikevich neuron model^45^. In this paper, we build on our previous work and evaluate the signal responses in CR, and compare the characteristics across these chaotic states through two methods. We first examine the dependence of the signal response on the maximum Lyapunov exponent; then we identify the resonant zone in the parameter space of the applied signal frequency/amplitude.

## Materials and Methods

### Izhikevich neuron model

The Izhikevich neuron model^36, 37^ is a two-dimensional ordinary differential equation of the form

1 $$\dot{v}=0.04{v}^{2}+5v+140-u+I,$$

2 $$\dot{u}=a(bv-u),$$

and with auxiliary after-spike resetting

3 $${\rm{if}}\,v\,\ge 30[{\rm{mV}}],{\rm{then}}\{\begin{array}{l}v\leftarrow c\\ u\leftarrow u+d\mathrm{.}\end{array}$$

Here, *v* and *u* represent the membrane potential of a neuron and the membrane recovery variable, respectively.

We extended Eq. (1) using a weak periodic signal *I*_ext_(*t*) as follows:

4 $$\dot{v}=0.04{v}^{2}+5v+140-u+I+{I}_{{\rm{ext}}}(t),$$

in which we adopted *I*_ext_(*t*) = *A*sin(2*πf*_0_ *t*). Note that the sinusoidal signal was utilized merely as a typical example of a signal in a neural system.

### Evaluation indices

#### Indices for evaluation of chaos and bifurcation

To quantify the chaotic activity in the Izhikevich neuron model, the Lyapunov exponent with a saltation matrix is utilized. On a system with a continuous trajectory between the *i*-th and the (*i* + 1)-th spiking times, (*t*_*i*_ ≤ *t* ≤ *t*_*i*+1_), the variational equations (1) and (2) are defined as follows:

5 $${\dot{{\rm{\Phi }}}}_{i+1}(t,{t}_{i})=J(v,u,t){{\rm{\Phi }}}_{i+1}(t,{t}_{i}),$$

6 $${{\rm{\Phi }}}_{i+1}({t}_{i},{t}_{i})=E,$$

where Φ, *J*, and *E* indicate the state transition matrix, the Jacobian matrix, and a unit matrix, respectively. At *t* = *t*_*i*_, the saltation matrix is given by

7 $${S}_{i}=[\begin{array}{cc}\frac{{\dot{v}}^{+}}{{\dot{v}}^{-}} & 0\\ \frac{{\dot{u}}^{+}-{\dot{u}}^{-}}{{\dot{v}}^{-}} & 1\end{array}],$$

In the above, (*v* ^−^, *u* ^−^) and (*v* ^+^, *u* ^+^) represent the values of (*v*, *u*) before and after spiking, respectively. In case spikes arise in the range [*T* ^*k*^:*T* ^*k*+1^] [ms], Φ^*k*^(*T* ^*k*+1^, *T* ^*k*^) (*k* = 0, 1, …, *N* − 1)^43^ can be expressed as

8 $${{\rm{\Phi }}}^{k}({T}^{k+1},{T}^{k})={{\rm{\Phi }}}_{i+1}({T}^{k+1},{t}_{i}){S}_{i}{{\rm{\Phi }}}_{i}({t}_{i},{t}_{i-1})\cdots {S}_{2}{{\rm{\Phi }}}_{2}({t}_{2},{t}_{1}){S}_{1}{{\rm{\Phi }}}_{1}({t}_{1},{T}^{k})\mathrm{.}$$

Based on the eigenvalues ${l}_{j}^{k}$ (*j* = 1, 2) of Φ^*k*^(*T* ^*k*+1^, *T* ^*k*^), the Lyapunov spectrum *λ*_*j*_ is calculated by

9 $${\lambda }_{j}=\frac{1}{{T}^{N}-{T}^{0}}\sum _{k=0}^{N-1}\,\mathrm{log}(|{l}_{j}^{k}|).$$

In our simulation, we set *T* ^*k*+1^ − *T* ^*k*^ as the time required for 20 spikes (*i* = 20). We set 1000 [ms] as the maximum value in case *T* ^*k*+1^ − *T* ^*k*^ lasts for 1000 [ms] before 20 spikes occur.

In order to conduct bifurcation analysis in the system with a state-dependent jump, we set a Poincar*é* section Ψ(*v* = 30). The dynamics of system behavior on Ψ are indicated by the Poincar*é* map *u*_*i*+1_ = *ψ*(*u*_*i*_) where *u*_*i*_ is the value of *u* on Ψ. In the literature^42^, the stability of a fixed point *u*_0_ = *ψ*(*u*_*l*−1_)$\cdots$ *ψ*(*u*_1_)*ψ*(*u*_0_) ≡ *ψ* ^*l*^(*u*_0_) (*l* = 1, 2, $\cdots$) is evaluated by

10 $${\mu }^{l}=\frac{\partial {\psi }^{l}}{\partial {{\bf{u}}}_{0}}=(\begin{array}{ll}0 & 1\end{array})(\begin{array}{cc}0 & 0\\ -\dot{v}/\dot{u} & 1\end{array}){\rm{\Phi }}({t}_{l},{t}_{0})(\begin{array}{c}0\\ 1\end{array}).$$

Here, **u**_0_ = (*v*_0_, *u*_0_) indicates the initial value of orbit **u** = (*v*, *u*) at *t* = *t*_0_. |*μ* ^*l*^ < 1|, *μ* ^*l*^ = −1, and *μ* ^*l*^ = 1 represent the stable condition, period-doubling bifurcation, and tangent bifurcation, respectively.

#### Indices for evaluation of signal response

We calculated the timing of the spikes against signal *I*_ext_(*t*) by using a cycle histogram $F(\tilde{t})$ ^33^. $F(\tilde{t})$ was a histogram of firing counts at *t*_*k*_ mod (*T*_0_) (*k* = 1, 2, …) against signal ${I}_{{\rm{ext}}}(\tilde{t})$ with period *T*_0_ = (1/*f*_0_), $0\le \tilde{t}\le {T}_{0}$. For example, for *T*_0_ = 10, if the spike times were *t*_*k*_ = 2, 6, 12, 16, 26, the values of *t*_*k*_ mod (*T*_0_) were 2, 6, 2, 6, 6. The cycle histogram then became *F*(2) = 2 and *F*(6) = 3.

To quantify the signal response, we used the following index of Eqs (11) and (14). The mutual correlation *C*(*τ*) between the cycle histogram $F(\tilde{t})$ of the neuron spikes and the signal ${I}_{{\rm{ext}}}(\tilde{t})$ is given by

11 $$C(\tau )=\frac{{C}_{IF}(\tau )}{\sqrt{{C}_{II}{C}_{FF}}}$$

12 $${C}_{IF}(\tau )=\langle ({I}_{{\rm{ext}}}(\tilde{t}+\tau )-\langle {I}_{{\rm{ext}}}(\tilde{t})\rangle )(F(\tilde{t})-\langle F(\tilde{t})\rangle )\rangle $$

13 $${C}_{II}=\langle {({I}_{{\rm{ext}}}(\tilde{t})-\langle {I}_{{\rm{ext}}}(\tilde{t})\rangle )}^{2}\rangle $$

14 $${C}_{FF}=\langle {(F(\tilde{t})-\langle F(\tilde{t})\rangle )}^{2}\rangle $$

For the time delay factor *τ*, we checked max_*τ*_ *C*(*τ*), i.e., the largest *C*(*τ*) between 0 ≤ *τ* ≤ *T*_0_.

## Results

### Parameter region for evaluating signal responses

**Figure 1:**
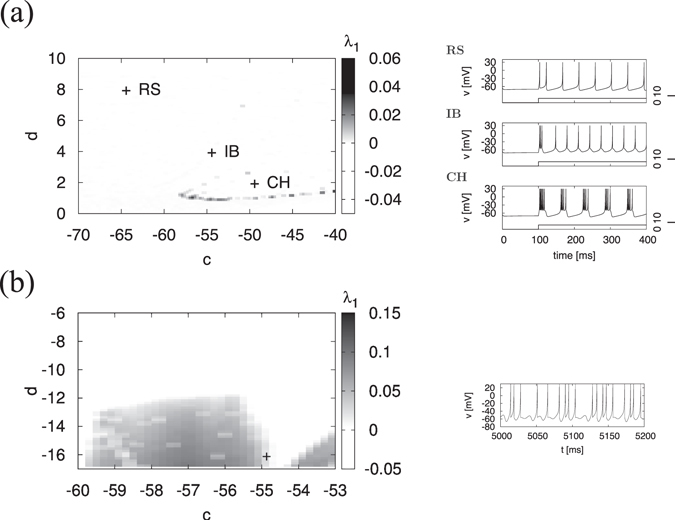
Dependence of maximum Lyapunov exponent *λ*_1_ on parameters *c* and *d*. (**a**) Region around the parameter sets for regular spiking (RS), intrinsically bursting (IB), and chattering (CH). The symbols of (+) indicate the parameter sets for RS and IB, CH (*a* = 0.02, *b* = 0.2, *I* = 10). The chaotic states (*λ*_1_ > 0) exist in −59 ≲ *c* ≲ −40, *d* ≈ 1.0. (**b**) Region around the parameter set proposed by Izhikevich for chaotic spiking. The symbols of (+) indicate the parameter set for chaotic spiking (*a* = 0.2, *b* = 2, *I* = −99). The chaotic states (*λ*_1_ > 0) exist in *d* ≲ −13.

**Figure 2:**
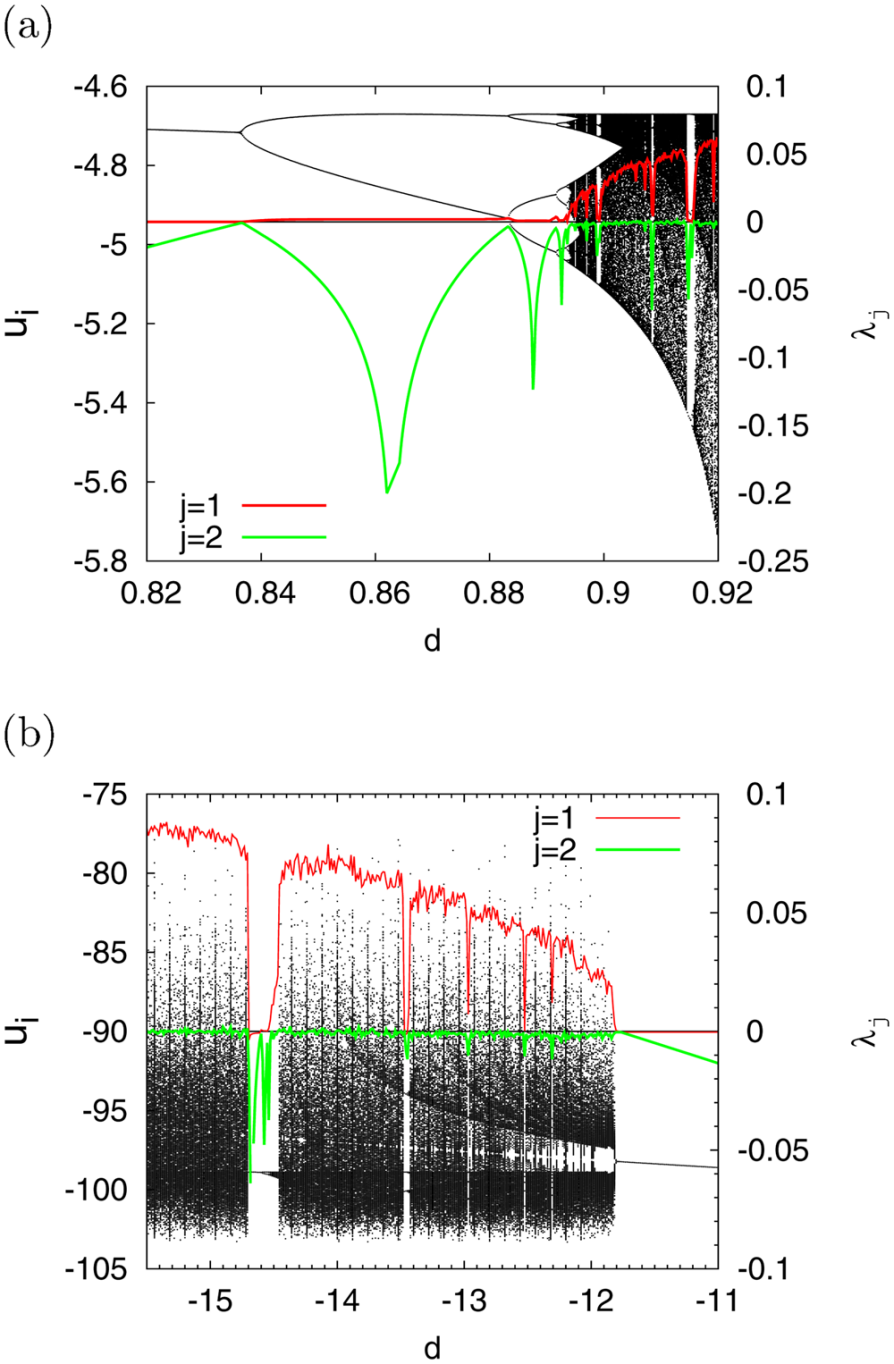
Bifurcation diagram of *u*_*i*_ on Poincar*é* section Ψ and Lyapunov exponents *λ*_*j*_ as function of parameter *d* (*j* = 1, 2). (**a**) Period-doubling bifurcation case (called region #1) (*a* = 0.02, *b* = 0.2, *c* = −55, *I* = 10). The period-doubling bifurcation (*μ* ^*l*^ = −1) arises at *d* ≈ 0.8348, 0.8828, 0.8916, 0.894, and the chaotic state (*λ*_1_ > 0, *λ*_2_ = 0) appears *d* ≳ 0.894 through a period-doubling bifurcation route. (**b**) Tangent bifurcation case (called region #2) (*a* = 0.2, *b* = 2, *c* = −56, *I* = −99). The tangent bifurcation (*μ* ^*l*^ = 1) arises at *d* ≈ −11.9 and the chaotic state (*λ*_1_ > 0, *λ*_2_ = 0) appears *d* ≲ −11.9 through the intermittency route.

Initially, we determined the parameter regions where the chaotic state is produced. The left panels of Fig. 1(a) and (b) show the dependences of the maximum Lyapunov exponent *λ*_1_ on parameters *c* and *d* in the region around parameter sets for the spiking patterns of RS, IB, and CH (see the right part of Fig. 1(a)) and the region around the parameter set proposed by Izhikevich for chaotic spiking (see the right part of Fig. 1(b)), respectively. The chaotic states (*λ*_1_ > 0) exist in −59 ≲ *c* ≲ −40, *d* ≈ 1.0 in the former case, and *d* ≲ −13 in the latter case. As the parameter regions for evaluating CR, we chose 0.82 ≤ *d* ≤ 0.92 in the former region (called region #1 below), and −15.5 ≤ *d* ≤ −11 in the latter region (called region #2 below). Figure 2 depicts the bifurcation diagram of *u*_*i*_ on Poincar*é* section Ψ (black dot) and Lyapunov exponents (red dotted (*j* = 1) and green dashed (*j* = 2) lines) as a function of parameter *d* in region #1 case (a) and region #2 case (b). In Fig. 2(a), the period-doubling bifurcation (*μ* ^*l*^ = −1) arises at *d* ≈ 0.8348, 0.8828, 0.8916, 0.894, and the chaotic state (*λ*_1_ > 0, *λ*_2_ = 0) appears *d* ≳ 0.894. Hence, the period-doubling bifurcation route to chaos exists in this region. However, as shown in Fig. 2(b), the tangent bifurcation (*μ* ^*l*^ = 1) arises at *d* ≈ −11.9 and the chaotic state *d* ≲ −11.9 (*λ*_1_ > 0, *λ*_2_ = 0) appears. This chaotic state produced by tangent bifurcation indicates the alternating laminar and turbulent modes of intermittency chaos in a general way^46^; this dynamic was demonstrated in our previous work^34^. That is, the intermittency route to chaos exists in this region.

### Signal response in chaotic resonance

**Figure 3:**
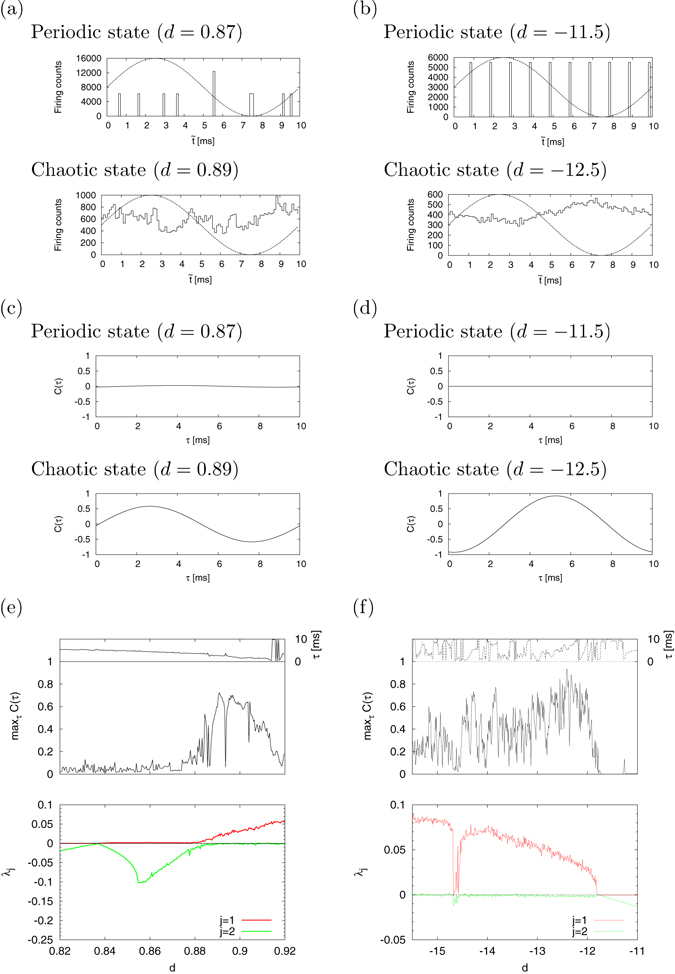
Dependence of signal response on parameter *d*. The cycle histogram $F(\tilde{t})$ of neuron spikes is congruent with the signal ${I}_{{\rm{ext}}}(\tilde{t})$ in chaotic regions, i.e., chaotic resonance (CR) arises. (**a**) Cycle histogram $F(\tilde{t})$ in the periodic state (upper) and the chaotic state (lower) in the case of region #1. (*a* = 0.02, *b* = 0.2, *c* = −55, *I* = 10). (**b**) The case of region #2. (*a* = 0.2, *b* = 2, *I* = −99). (**c**) Mutual correlation *C*(*τ*) between the cycle histogram $F(\tilde{t})$ of the neuron spikes and the signal ${I}_{{\rm{ext}}}(\tilde{t})$ in the case of region #1. (**d**) The case of region #2. (**e**) max_*τ*_ *C*(*τ*) (upper) and Lyapunov exponent *λ*_*j*_ (*j* = 1, 2) (lower) as a function of parameter *d* in the case of region #1. (**f**) The case of region #2.

In the above mentioned chaotic parameter regions #1 and #2, we evaluated the response against a weak signal (*A* = 10^−2^, *f*_0_ = 0.1). To begin with, we compared the cycle histograms $F(\tilde{t})$ between periodic and chaotic states. As shown in Fig. 3, in the cases of both region #1 (a) and region #2 (b), $F(\tilde{t})$ in the periodic state (solid line) does not fit ${I}_{{\rm{ext}}}(\tilde{t})$ (dotted line) because the periodic response against ${I}_{{\rm{ext}}}(\tilde{t})$ induces growth in its values at specific bins. On the other hand, $F(\tilde{t})$ in the chaotic state fits ${I}_{{\rm{ext}}}(\tilde{t})$ according to a chaotic response with scatter timing against *I*_ext_(*t*). This tendency can also be observed in their *C*(*τ*) as shown in Fig. 3(c) and (d). That is, *C*(*τ*) becomes approximately 0 in the periodic state, but *C*(*τ*) exhibits a sinusoidal shape in the chaotic state. In the following evaluations, we use max_*τ*_ *C*(*τ*) to characterize the signal response, because the sinusoidal shape of *C*(*τ*) with period *T*_0_ can be identified by amplitude and lag corresponding to ${\max }_{\tau }C(\tau )$ and its *τ* value. Furthermore, this signal response is evaluated using ${\max }_{\tau }C(\tau )$ and *λ*_1_. Figure 3(e) and (f) show the dependence of ${\max }_{\tau }C(\tau )$ (upper) and *λ*_*j*_ (*j* = 1, 2) (lower) on parameter *d* in regions #1 and #2, respectively. In region #1 (Fig. 3(a)), the neuron exhibits the periodic spiking (*λ*_1_ ≈ 0, *λ*_2_ < 0) in 0.82 ≲ *d* ≲ 0.88, and the chaotic spiking (*λ*_1_ > 0, *λ*_2_ ≈ 0) in 0.88 ≲ *d* ≲ 0.92. In the periodic spiking state, the value of ${\max }_{\tau }C(\tau )$ is less than 0.1; whereas in the chaotic spiking state, the value of ${\max }_{\tau }C(\tau )$ is higher in comparison with the periodic spiking state. In particular, at the *d* ≈ 0.89 location around the bifurcation to chaos, called the edge of chaos^47^ below, ${\max }_{\tau }C(\tau )$ has a peak value (≈0.8). This can be interpreted as CR arising in the chaotic region (0.88 ≲ *d* ≲ 0.92). In region #2 (Fig. 3(b)), the chaotic spiking state (*λ*_1_ > 0, *λ*_2_ ≈ 0) arises in −15.5 ≲ *d* ≲ −12 and ${\max }_{\tau }C(\tau )$, and is a high value due to the effect of this chaotic spiking state. Also, the value of ${\max }_{\tau }C(\tau )$ indicates a similar tendency for region #1 (Fig. 3(a)), i.e., at the *d* ≈ −12.3 location around the bifurcation to chaos, ${\max }_{\tau }C(\tau )$ has a peak value (≈0.9).

**Figure 4:**
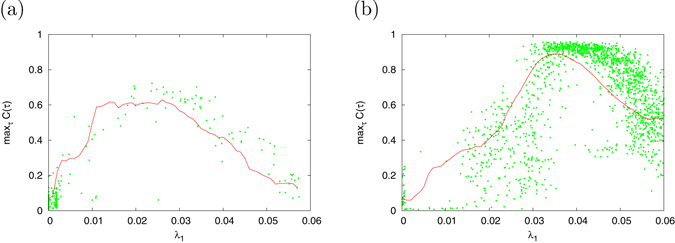
Scatter plot between max_*τ*_ *C*(*τ*) and *λ*_1_ obtained in Fig. 3 (red dotted line: mean value of max_*τ*_ *C*(*τ*) in bin *λ*_1_ with window Δ*λ*_1_ = 0.001). Signal response in CR has a unimodal maximum with respect to the stability for chaotic orbits represented by *λ*_1_, (**a**) Region #1. (**b**) Region #2.

Furthermore, Fig. 4(a) and (b) show the scatter plots between ${\max }_{\tau }C(\tau )$ and *λ*_1_ obtained in Fig. 3 in the cases of region #1 and region #2, respectively. The red dotted line indicates the mean value of ${\max }_{\tau }C(\tau )$ in bin *λ*_1_ with window Δ*λ*_1_ = 0.001. From these results, in both regions, ${\max }_{\tau }C(\tau )$ peaks at the appropriate value of max_*τ*_ *C*(*τ*) (${\max }_{\tau }C(\tau )\approx 0.7$ at *λ*_1_ ≈ 0.03 in region #1 and ${\max }_{\tau }C(\tau )\approx 0.9$ at *λ*_1_ ≈ 0.04 in region #2). The points for this appropriate value for *λ*_1_ correspond to the points representing the edge of chaos in Fig. 3. That is, the signal response in CR has a unimodal maximum with respect to the stability for chaotic orbits represented by *λ*_1_, and this peak is localized at the edge of chaos.

### Signal sensitivity in the edge of chaos region

**Figure 5:**
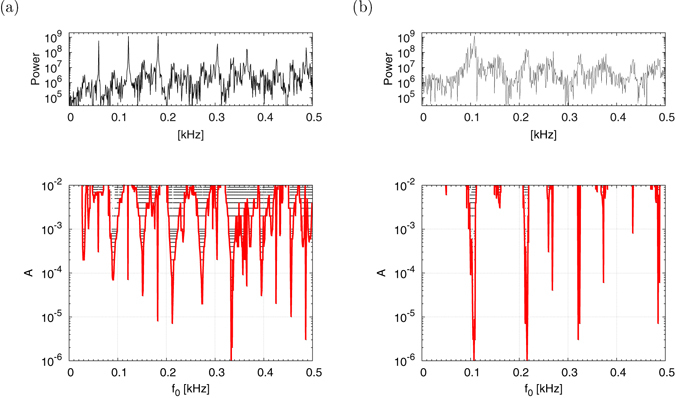
Power spectrum of the time series of *v*(*t*) in the signal-free condition (upper). Resonant frequency *f*_0_/amplitude *A* zone and points (max_*τ*_ *C*(*τ*) > 0.5), indicated by the red line and black points, respectively, in the signal-adapted condition (lower). The resonant zones have a tendency to distribute near the peaks for the power spectrum in the signal-free condition. (**a**) Edge of the chaotic state in region #1 (*d* = 0.896). (**b**) Edge of the chaotic state in region #2 (*d* = −12.0).

In the edge of chaos, i.e., the chaotic state near the bifurcation point, the power spectrum for the time series of system behavior has several peaks. In the periodic bifurcation route to chaos, the trajectory is restricted to the narrow space around the multiple-periodic trajectory before the points at the bifurcation to chaos. Therefore, the power spectrum of the chaotic state inherits the peaks from the power spectrum of the multiple-periodic state, while in the intermittency route to chaos, the laminar state dominates in the time series of system behavior. Hence, the power spectrum has peaks near the frequency components of the laminar state. The upper panels of Fig. 5 show the power spectrum of *v*(*t*) under the signal-free condition in the edge of chaos in region #1 (a) (*d* = 0.896) and #2 (b) (*d* = −12.0). For the reasons described above, the power spectrum has several peaks. Furthermore, as shown in the lower panels of Fig. 5, resonant frequency/amplitude zones and points (max_*τ*_ *C*(*τ*) > 0.5) indicated by the red line and black points, respectively, in the signal-adapted condition. Here, its frequency *f*_0_ corresponds to the horizontal line of the upper panels of Fig. 5. From this result, the resonant zones have a tendency to distribute near the peaks for the power spectrum in the signal-free condition. This is especially significant with the weaker signal amplitude regions.

## Discussion and Conclusions

We showed herein two distinct routes to chaos, the period-doubling bifurcation route and the intermittency route, by using the Lyapunov exponent with a saltation matrix and index for stability of a fixed point on the Poincaré section. Furthermore, under the condition of receiving input from a weak periodic signal, the enhancement of the signal response by the effect of chaotic spikes (chaotic resonance) was confirmed in the chaotic regions induced by the above routes to chaos. Specifically, in both chaotic regions, the signal response in CR had a unimodal maximum with respect to the stability for chaotic orbits represented by *λ*_1_. Thus, it can be interpreted that the instability of the chaotic orbit in CR plays a role of the noise strength in SR.

Furthermore, we have confirmed that the peak of the signal response was located in the edge of chaos. There, we identified the periodic components in chaotic spiking activity as shown in Fig. 5. In the case of a relatively large signal strength, we found broadening of the signal frequency region in which the efficient signal response was high. On the other hand, in the case of a small signal strength, the region of high efficiency was restricted to the immediate neighborhoods of frequencies for the periodic components in chaotic spiking. This characteristic of signal response in relation to signal strength and frequency, called Arnold’s tongue, is widely observed in synchronization phenomena^48^. Therefore, the high efficiency of signal responses in the edge of chaos could be interpreted as synchronization between the input signal and the periodic component in chaotic spiking activity.

In future work, we intend to evaluate the signal response in CR in large-sized spiking neural networks.
